# Robot Assisted Treatment of Hand Functional Rehabilitation Based on Visual Motor Imagination

**DOI:** 10.3389/fnagi.2022.870871

**Published:** 2022-04-08

**Authors:** Long Li, Yanlong Zhang, Liang Huang, Jie Zhao, Jue Wang, Tian Liu

**Affiliations:** ^1^The Key Laboratory of Biomedical Information Engineering of Ministry of Education, School of Life Sciences and Technology, Xi’an Jiaotong University, Xi’an, China; ^2^The Key Laboratory of Neuro-Informatics and Rehabilitation Engineering of Ministry of Civil Affairs, Institute of Health and Rehabilitation Science, School of Life Sciences and Technology, Xi’an Jiaotong University, Xi’an, China; ^3^National Engineering Research Center of Health Care and Medical Devices, Xi’an, China; ^4^Key Laboratory for Artificial Intelligence and Cognitive Neuroscience of Language, Xi’an International Studies University, Xi’an, China

**Keywords:** steady-state visual evoked potential (SSVEP), motor imagery (MI), inter trial phase locking consistency (ITPC), robot assisted therapy, electroencephalogram

## Abstract

This pilot study implements a hybrid brain computer interface paradigm based on motor imagery (MI) and steady-state visual evoked potential (SSVEP), in order to explore the neural mechanism and clinical effect of MI-SSVEP intervention paradigm on upper limb functional rehabilitation. In this study, EEG data of 12 healthy participants were collected, and the activation regions of MI-SSVEP paradigm were identified by power spectral density (PSD). By analyzing the inter trial phase consistency (ITPC) of characteristic regions and the causal relationship of brain network, the motor cognitive process including high-level somatosensory joint cortex in the intervention process of MI-SSVEP was studied. Subsequently, this study verified the clinical effect of MI-SSVEP intervention paradigm for 61 stroke patients. The results show that the robot assisted therapy using MI-SSVEP intervention paradigm can more effectively improve the rehabilitation effect of patients.

## Introduction

Stroke is one of the leading causes of motor dysfunction and death worldwide. It affects more than 17 million people every year, and most stroke patients have motor dysfunction, which seriously affects the daily life of stroke survivors (Uzdensky, 2020). Due to the existence of brain neural plasticity (Hummel and Cohen, 2005), many studies have found that patients’ motor ability can be significantly restored through rehabilitation training (Becerra-Calixto and Cardona-Gomez, 2017). This phenomenon supports most repetitive, goal-based rehabilitation practices, because imitating natural motion patterns may lead to rewiring and strengthening motor neural networks to restore normal motor function. Therefore, how to “mobilize” the brain to produce similar natural movement patterns is the main goal of clinical rehabilitation of stroke.

Robot assisted rehabilitation therapy is one of the widely used interventions in clinical stroke rehabilitation therapy (Jihun and Jaehyo, 2017). With the development of brain science, the brain-computer interface method based on visual stimulation has gradually entered clinical robot assisted rehabilitation training (Grimm et al., 2016), and the rehabilitation therapy aimed at improving patients’ willingness to actively recover has gradually entered clinical applications, and previous studies have shown that the effect of traditional passive robot assisted rehabilitation training is not significant, compared with active rehabilitation training (Yang et al., 2021). At present, the brain-computer interface paradigm used in most clinical trials involves two types of methods, steady state visual evoked (SSVEP) and motor imagery (MI) (Stefano Filho et al., 2021).

Steady state visual evoked refers to visual periodic stimulation with a certain frequency, and the EEG of cerebral cortex also contains signal characteristics with the same frequency, which is referred to as SSVEP for short (Cheng et al., 2002). Light arrives at the retina through the human eye structure to form an image. This image information is transmitted to the visual cortex of the nerve center through the optic nerve. The change of ganglion cell activity leads to the change of potential, so that regular EEG signals can be detected in the occipital lobe, the whole process of visual evoked and its physiological structure. At present, SSVEP is widely used in brain computer interface control in clinical robot assisted rehabilitation. In general, the process of steady-state visual induction is closer to the human body’s own neural function response, and has less correlation with active intention.

The physiological basis of motor imagery (MI) is due to the existence of mirror neurons in humans (Carrillo-de-la-Pena et al., 2008). Existing studies have shown that sensorimotor regions can be activated through motor imagination tasks (Hummel and Cohen, 2005; Rizzolatti and Craighero, 2004). At the same time, the motor imagination task will also activate the contralateral brain region, improve the blood flow and oxygen supply, and produce the phenomenon of desynchronization, while the ipsilateral brain energy increases and the synchronization is enhanced (Li et al., 2015). For stroke patients, motor imagery can activate mirror neurons in the contralateral motor area when the nerves in the motor area of the brain are damaged or the neuronal connections are abnormal (Figueroa-Garcia et al., 2014). Meanwhile, repeated activation of contralateral motor area neurons can promote nerve germination and nerve facilitation (Kim and Choe, 2009). However, motor imagery needs long-term training, and it is difficult for untrained people to directly desynchronize (Maksimenko et al., 2018).

Although both SSVEP and MI are used in robot assisted rehabilitation, these two methods have some defects in clinical application. Firstly, although the traditional motor imagination can activate motor mirror neurons, its classification rate is not high and needs to be trained in advance. Some people can not obtain the ideal classification effect of motor imagination even through training (Sitaram et al., 2007). Secondly, training only through SSVEP method cannot effectively activate mirror neurons, which makes the actual rehabilitation effect not ideal. At the same time, after long-term induced stimulation, it is easy to lead to patients’ inattention and increase the degree of visual fatigue, thus affecting the rehabilitation effect (Amiri and Fazelrezai, 2013). During the clinical experiment, it is found that the rehabilitation effect is often better when the two methods are combined.. Therefore, some researchers have designed a hybrid brain computer interface paradigm based on MI-SSVEP and verified its feasibility. Mcgeady et al. (2019) used three different hand action photos to guide the motor imagination, and made one of the pictures flicker at a certain frequency to achieve the effect of mixed stimulation. Mcgeady et al. (2019) show that the system can be used for neural rehabilitation training. Similar studies show that compared with the simple brain computer interface of motor imagination, users using MI-SSVEP method can have higher resolution and robustness without motor imagination training. Previous studies on MI-SSVEP method mainly focused on the classification success rate of EEG signals. However, the neural mechanism of MI-SSVEP is not clear. At the same time, we believe that the previous paradigm of MI-SSVEP method still has defects and needs to be further improved. Static arrows or pictures and other guidance methods cannot well induce the occurrence of motion imagination, while dynamic motion animation can often better induce its occurrence (Yao et al., 2015). In this study, we designed an active training program of motor task steady-state visual evoked (MI-SSVEP) hand function, hoping to study the effect of MI-SSVEP stimulation paradigm through this program, and verify whether this paradigm can effectively affect the motor function of participants. We hypothesized that MI-SSVEP stimulation paradigm could activate major brain regions related to motor function and change the activation pattern of the brain in motor tasks. Firstly, we used normal subjects to observe the brain power spectral density distribution under the intervention of MI-SSVEP paradigm and the consistent spectral distribution between active brain regions. At the same time, we observed the causal connection between different brain regions to analyze the neural mechanism of MI-SSVEP intervention paradigm on the rehabilitation of upper limb function. Then we used this paradigm to verify the clinical effect, and followed 84 stroke patients with rehabilitation training.

## Materials and Methods

### Participants

#### Neural Mechanism Validation Participants

Twelve healthy right-handed volunteers (average age 22 years; range 18–21 years; five men and seven women) who had no depression or other types of neurological diseases, and no history of medication for mental illness. Before the start of the experiment, they were not informed of the purpose, intention, and detailed process of the experiment. None of the 12 subjects had undergone motor imagination training in advance. There was no significant difference in the average Fugl-Meyer (FMA-UE hand function test item) scores of the 12 subjects. All subjects signed an informed consent form, which was approved by the ethics committee of Xi’an Jiaotong University.

#### Clinical Trial Participants

The inclusion criteria of rehabilitation training were the patients with hand motor dysfunction after stroke, the length of illness was within six months, no shoulder dislocation, no cognitive impairment, and the age was between 30 and 70 years old. There were 84 patients in total. We divided the subjects into two groups with 42 people in each group. Due to the intervention design, the inclusion and exclusion criteria of patients are as follows:

Inclusion criteria: (1) Between 30 and 70 years old; (2) Unilateral stroke with onset time less than 6 months (based on the results of head CT or MRI); (3) The neuro-nutrition, drugs to promote the recovery of brain function and daily rehabilitation methods taken by stroke patients were similar.

Exclusion criteria: (1) History of previous stroke; (2) Neuroradiological evidence of hemispheric involvement; (3) Dementia or aphasia is serious enough to affect the patient’s compliance with the experiment; (4) In addition to stroke, there are other neurological diseases; (5) The hands cannot complete subtle flexion movement, that is, the EMG signal cannot be recorded when performing the experimental task (this article is only applicable to the exclusion criteria in the second part of the experiment).

All subjects signed informed consent and were approved by the ethics committee of Xi’an Jiaotong University.

### Experimental Paradigm

#### Neural Mechanism Validation Paradigm

**FIGURE 1:**
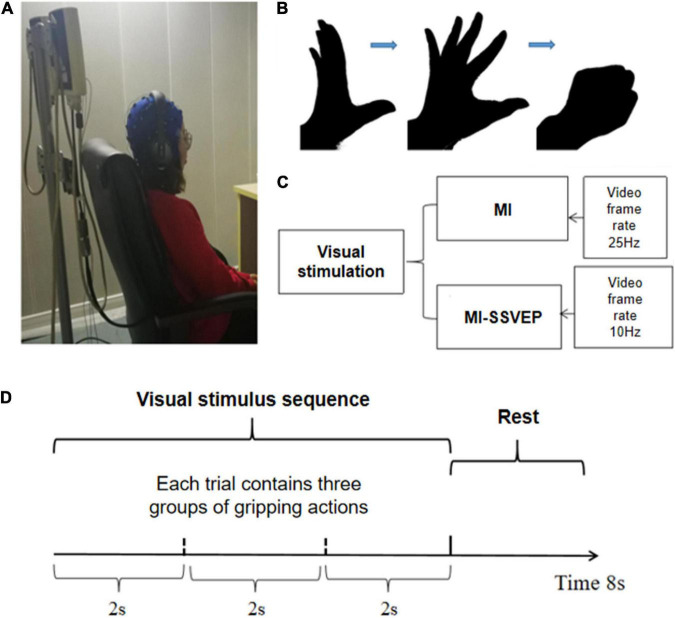
Task design. **(A)** The picture of the experimental environment, the subjects kept sitting relaxed with their hands flat on their legs. **(B)** The screen plays the animation of grasping action stimulation. The whole grasping action is divided into 50 pictures and played continuously in two seconds; **(C)** The experimental stimulation group was divided into MI stimulation group and MI-SSVEP stimulation group. The MI-SSVEP stimulation group added 10 Hz strobe when playing animation; **(D)** Each trial has a total of 8 s, and a total of three complete grasping movements are played during the stimulation time.

The MI-SSVEP experiment combines motor imagination and visual evoker to give the subjects visual guidance on grasping actions. The stimulation contains frequency information and action guidance. Frequency stimulation can induce the subjects’ occipital area of the visual cortex EEG to appear related frequencies information. Motion guidance can activate mirror neurons in the sensorimotor area. Visual stimuli in EEG are generally stable and robust, so this paradigm can complete the collection of highly robust EEG signals that activate motor mirror neurons. In order to study the difference between MI and MI-SSVEP, this article selects the grasping action as the goal of motor imagination task. The specific experimental process is as follows: the subject sits on a chair, maintains a comfortable sitting posture and eyes level, the front chest is about 30 cm away from the desktop, and the screen resolution used for visual stimulation is 60 Hz. The EEG acquisition device uses a 32-lead Neuroscan EEG acquisition device. Before the experiment, make sure that the impedance of all leads is below 5 KΩ, and check whether the EEG signal amplitude and rhythm are normal. The whole experiment contains two groups of experiments, each group contains 80 trials, a trial has a total duration of 8 s, and each trial contains 2 s of rest time and 6 s of stimulation time. The MI group stimulus pictures are black and white hand grasping movements, and the MI-SSVEP stimulus pictures are also black and white hand grasping movements. During the stimulation process, the picture will flash at a frequency of 10 Hz, and the rest time of each group of stimulation is displayed. In the picture, REST is marked with black font on the image for three seconds. The screen background is gray, the foreground of the stimulus image is black, and the background is white. The matching of black and white is to give the subjects the maximum color visual difference. All 12 subjects received MI stimulation and MI-SSVEP stimulation, and the order of stimulation was given in a random alternating manner. The detailed experimental process is shown in Figure 1.

#### Clinical Trial Paradigm

In the clinical experiment, in order to verify whether the MI-SSVEP method can effectively improve the traditional hand function robot assisted therapy, we divided the subjects into two groups with 42 people in each group. Group A was treated with manual robot assisted therapy based on MI-SSVEP intervention paradigm, and group B was treated with hand functional robot assisted therapy. Both groups received two hours of upper and lower limb training every day, six times a week. Base on routine treatments, the patients of the two groups will increase intervention training three times a week. Each intervention training lasts for 30 min, with a rest adjustment of about one minute every 5 min. Each person continued training for 2 weeks and made tracking records.

### Recordings

All experiments are carried out in electromagnetic shielding room. EEG data was recorded using SynAmps2 system (EI Paso Neuroscan). According to the extended 10–20 system, a flexible cap with 32 Ag/AgCI electrodes is located. This system is usually used to detect brain electrical activity records with the parietal lobe as a reference. The electrode impedance is kept below 5 KΩ. All data are based on the electrode centered between Fz and Cz. EEG data is band-pass filtered (0.5–100 Hz). All signals are sampled to the hard disk at a frequency of 1,000 Hz, and stored on the hard disk together with event markers for offline analysis.

### Data Analysis

First, we use Neuroscan’s EDIT4.3 to preprocess the original EEG data, mainly using 50 Hz notch filter and ocular wake removal. In order to capture the differences between the prefrontal cortex, the motor sensory cortex, and the occipital visual cortex under different stimulation paradigms, the power spectrum analysis, time-frequency phase coherence and brain network connection analysis of all EEG channels were performed. The main analysis work in the research was carried out in the Matlab environment. Our analysis program was written using an open-source toolbox Field-Trip, and neurophysiological data analysis was carried out through this program.

### Power Estimated of EEG

This chapter uses the multi-cone method to calculate the power spectrum of the EEG signal, and normalizes the power of the EEG to the sum of the total power of each frequency (0–50 Hz). This calculation is an estimate of the power ratio for the main research frequency band. In the calculation process, the EEG electrodes corresponding to the brain regions of this study were selected. They include: left motor function cortex (lM), right motor function cortex (rM), somatosensory motor cortex (fM), occipital visual cortex (oM). The calculated data segment only includes the 6-s period during which visual stimulation occurs, and the EEG during the rest period is not included in the analysis process.

### Inter-Trial Coherence Calculation

Observing the desynchronization and synchronization of EEG signals is an important way to judge the overall effect of motor imagination. The synchronization and desynchronization effects can be viewed intuitively and comprehensively by using the time-frequency analysis method. Most research on hearing uses ITPC to calculate the correlation between signals, but for visual stimuli, ITLC can preserve the linear correlation between signals, more so, the two calculations in this chapter Calculated separately. First, a time-frequency analysis of the original data in EEGLAB was carried out one by one. And use the “newtimef” function to calculate inter-group correlation linear coherence (ITLC) and inter-group phase coherence (ITPC):

$$\text{ITLC}\,\left(f,t\right)=\frac{\sum_{i=1}^{n}F_{k}\,\left(f,t\right)}{\sqrt{n\,\sum_{i=1}^{n}{\left|F_{k}\,\left(f,t\right)\right|}^{2}}}$$

$$\text{ITPC}\,\left(f,t\right)=\frac{1}{n}\,\sum_{k=1}^{n}\frac{F_{k}\,\left(f,t\right)}{\left|F_{k}\,\left(f,t\right)\right|}$$

In this function, F_k_(f,t) is the spectral estimate of trial k at frequency f and time t obtained using short-time Fourier transformation (STFT), and || represents the complex norm of trial k. The modified STFT (with Hanning tapers) in EEGLAB uses overlapping sliding windows that are adaptive to the target frequency bins (i.e., the time window decreases linearly as frequency increases), which is recommended to overcome limitations of conventional fixed window in estimating low frequency activities. The frequency range analyzed was 0.5–50 Hz. Zero-padding was applied to windows without sufficient number of sample points with a pad-ratio of 16 with a frequency spacing of 0.5 Hz. ITPC value of a given frequency at a given time point can range from 0 to 1. Larger ITPC values indicate higher phase consistency across trials, and smaller values indicate lower consistency or larger neural “jittering.” For the calculation of theta ITPC, the ITPC data were first averaged across the frequencies for further processing.

### Convergent Cross Mapping

Identifying causal networks is important for dynamic mechanism for coordinating neural activity across neuronal networks and controlling the timing of neuronal firing. In this study, we use convergent cross mapping (CCM) to estimate causal relationship between sensorimotor area, frontoparietal lobe and visual cortex. CCM is a new method, based on nonlinear state space reconstruction, that can distinguish causality from correlation (Sugihara et al., 2012). The CCM result usually expressed as a normalized quantity between −1 and 1. The calculation process and method of CCM are described briefly below:The image space reconstruction is the first step in the non-linear dynamic analysis of EEG and EMG signals.*X*(*k*) and *Y*(*k*),*k* = 1,2,⋯*N* denotes the time series which we study. Its m-dimensional delay vector can be expressed as *X*(*n*) = {*x*(*n*),*x*(*n*−τ),*x*(*n*−2τ),⋯,*x*(*n*−(*m*−1)τ)}, *m* denotes embedding dimension, τ denotes time delay. *X*(*n*) denotes the reconstructed manifold *M*_*X*_. Next step is to generate a cross-mapped estimate of *Y*(n), denoted by *Y*⌢ (*n*)|*M*_*X*_.


$$\overset{⌢}{Y}\left(n\right)|M_{X}=\sum w_{i}\,Y\,\left(n_{i}\right)\,i=1\,\ldots \,m+1$$

The *w*_*i*_ is a weighting based on the distance between *x*(*n*) and its *i*th nearest neighbor on *M*_*X*_ and *Y*(n_i_) are the contemporaneous values of *Y*. The *w*_*i*_ can be expressed as

$$w_{i}=u_{i}/\sum u_{j}\,j=1\,\ldots \,m+1$$

where

$$u_{i}=\exp\left\{-d\,\left[\underline{x}\,\left(n\right),\underline{x}\,\left(n_{i}\right)\right]/d\,\left[\underline{x}\,\left(n\right),\underline{x}\,\left(n_{1}\right)\right]\right\}$$

$d\,\left[\underline{x}\,\left(n\right),\underline{x}\,\left(n_{i}\right)\right]$ is the Euclidean distance between two vectors.

The last step is to calculate the correlation coefficient ρbetween predicted and observed *X*.

### Clinical Application Validation

This part of the work is completed by Xi’an trade union hospital to evaluate and record all the tested data. The subjects were tested with Fugl-Meyer, Wolf and ARAT. After two weeks of intervention, the two groups were evaluated with the scale again and recorded statistically. Cases such as transfer, discomfort caused by high muscle tone and withdrawal from the experiment were excluded. Secondly, the EEG data is filtered (0.5–100 Hz) to manually remove the disturbed data segments such as eye electricity. After preprocessing, EEG data is converted into a data structure suitable for field trip toolbox through the built-in interface function between EEG lab toolbox and field trip toolbox for subsequent processing and calculation.

## Results

### Topographic Map of EEG Power Spectrum

**FIGURE 2:**
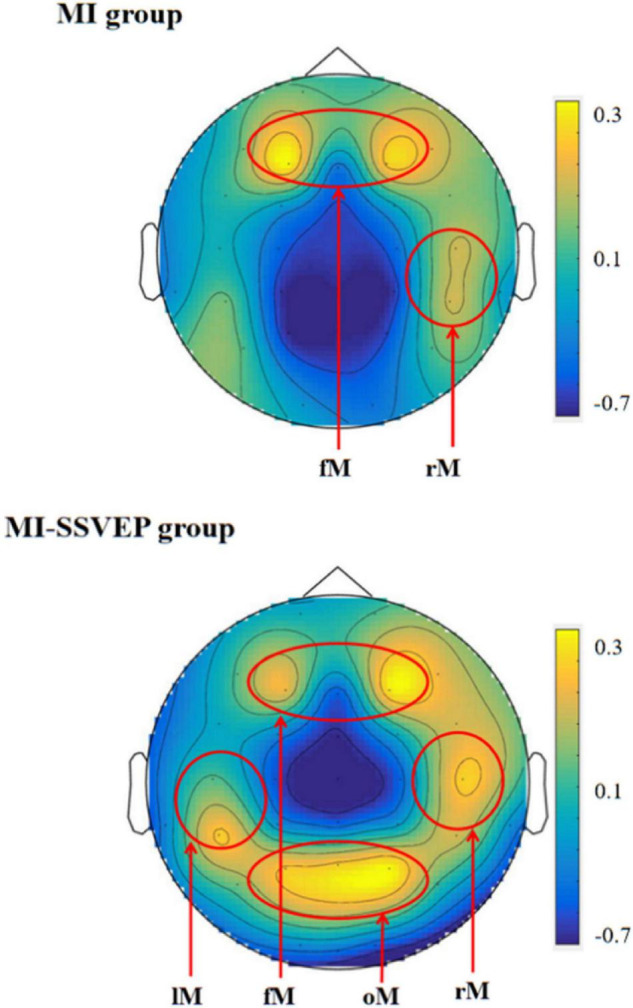
Mean EEG power spectrum of subjects.

In the experiment, we compared the distribution characteristics of EEG power and power spectrum under MI stimulation group and MI-SSVEP stimulation group to judge whether there is a differential distribution of brain activity under the traditional paradigm spontaneous motor imagination task and SSVEP induced motor imagination characters. During the analysis, we superimposed and averaged the data of 12 subjects in order to analyze the characteristics of brain activity under different tasks. As shown in Figure 2, we found that the main areas of brain activation in traditional motor imagination tasks are frontal parietal lobe (fM) and right motor cortex (rM). As shown in Table 1, the active areas of the brain in the motor imagination task guided by SSVEP were significantly different from those in the simple motor imagination task, and were significantly activated in the occipital lobe (oM) and left motor cortex (lM).

**TABLE 1:** The statistical analysis result of EEG power spectrum.

Area	*F*	df	*P*-value
fM	1.231	1	0.275
lM	6.274	1	0.017
rM	2.067	1	0.161
oM	5.408	1	0.031

*Frontal parietal lobe (fM), left motor cortex (lM), right motor cortex (rM), occipital lobe (oM).*

### Inter-Trial Phase Coherence

**FIGURE 3:**
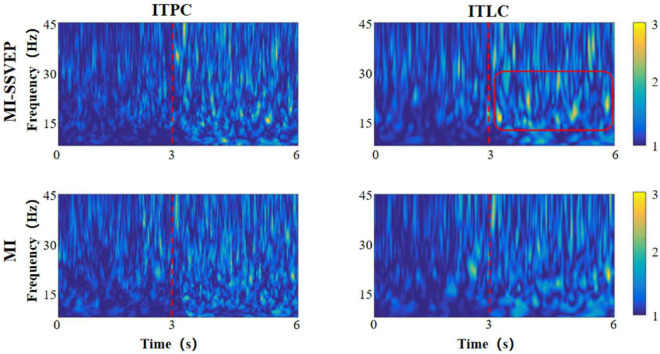
Time frequency distribution of frontal parietal ITLC / ITPC.

**FIGURE 4:**
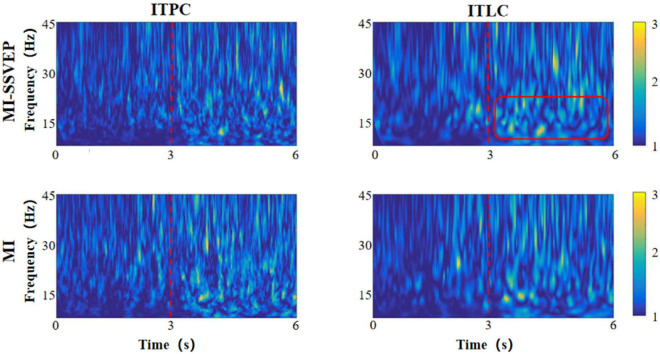
Time frequency distribution of ITLC / ITPC in left motor cortex.

**FIGURE 5:**
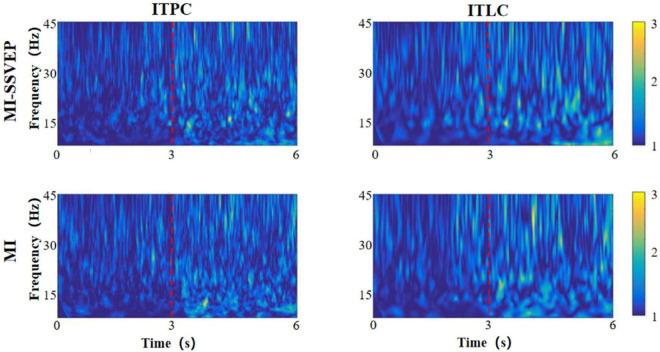
Time frequency distribution of ITLC / ITPC in right motor cortex.

**FIGURE 6:**
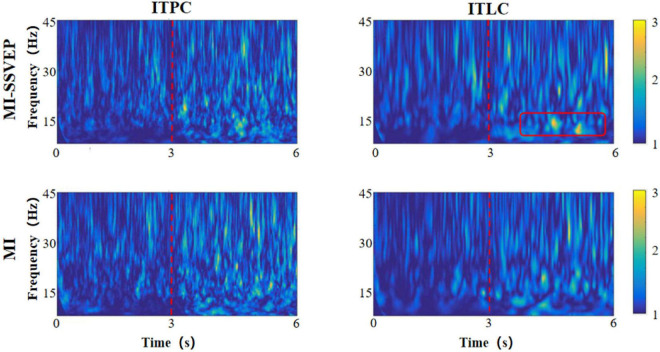
Time frequency distribution of ITLC / ITPC in occipital cortex.

As two time-frequency measurement methods, ITLC / ITPC observe the phase correlation and signal amplitude linear correlation between signals, respectively. ITLC / ITPC are widely used to study the relationship between EEG evoked potential and spontaneous EEG oscillation phase, and display the relevant cognitive processes in the brain. In this chapter, we analyzed the distribution characteristics of ITLC / ITPC in prefrontal lobe (Figure 3), left (Figure 4) and right parietal motor cortex (Figure 5) and occipital visual cortex (Figure 6).

It can be found from the figure that the consistency change caused by motion imagination has an obvious gradual process in time distribution. Whether the MI stimulation group or the MI-SSVEP stimulation group, the consistency of each brain region is at a low level within 1–3 s after the beginning of the experiment. The consistency of each brain region gradually increases within 3–6 s, and the consistency level reaches the highest when the stimulation lasts around 5 s. There are also some obvious differences in the consistent frequency domain distribution. In the stimulation of MI-SSVEP group in fM region, there is a high consistent distribution in the beta frequency band within 3–6 s, but there is no such feature in the MI stimulation group. In the lM region, both MI-SSVEP stimulation and MI stimulation showed a high consistent distribution in the beta band within 3–4 s, and then the consistent distribution of MI-SSVEP shifted to the low gamma band. In the rM region, the MI stimulation group had a significantly high consistent distribution in the gamma band. In the oM region, the alpha band of MI-SSVEP stimulation showed a significantly high consistent distribution in 4–6 s.

### Convergent Cross Mapping

The dynamic mechanism of neural activity coordination among neural networks during tactile stimulation was studied by using convergent cross mapping (CCM) analysis method. We selected the frontal parietal lobe (fM), left and right motor cortex (lM, rM) and occipital lobe (oM) as the main focus areas of CCM analysis, and calculated their two-way causality. The results are shown in Table 2.

**TABLE 2:** The statistical analysis result of CCM.

Group		Df	*F*	*P*-value
1	lM_fM	23	0.15	0.702
2	fM_lM	23	0.001	0.977
3	rM_fM	23	0.428	0.52
4	fM_rM	23	0.006	0.937
5	lM_oM	23	0.006	0.94
6	oM_lM	23	0.005	0.943
7	rM_oM	23	0.438	0.515
8	oM_rM	23	0.625	0.438
9	oM_fM	23	1.378	0.253
10	fM_oM	23	14.581	0.001

*Frontal parietal lobe (fM), left motor cortex (lM), right motor cortex (rM), occipital lobe (oM) “lm_fm” in the tableindicates the one-way causal connection from lM brain area to fM brain area, “fm_lm” indicates the one-way causal connection from fM brain area to lM brain area. Other expressions are similar.*

### Clinical Trial Results

In the clinical experiment, 84 patients were divided into two groups on average. Group A adopted the traditional clinical rehabilitation intervention method combined with manual robot assisted therapy using the “peripheral central peripheral” loop intervention paradigm, and group B adopted the traditional clinical rehabilitation intervention method and manual robot assisted therapy. Among them, 30 patients in group A and 31 patients in group B finally completed the experiment. We recorded the Fugl-Meyer, Wolf and ARAT scores of patients before and after two weeks of intervention.

We compared the differences between groups and within groups on the statistical results of group ab. the differences between groups mainly compared the differences between group A and group B, which were divided into two stages before and after the intervention. It should be emphasized that the grouped variables here are groups, and the compared variables are pre-test data and post-test data. In this section, in order to confirm whether the effect of the statistical experimental group is obvious, the difference between the two groups before and after intervention is also compared. The intra group difference is mainly compared with the control comparison before and after intervention in group A and group B. here is to compare the difference between pre-test and post-test in group A or group B. here, paired sample *t*-test is used. The statistical results are shown in Tables 3, 4.

**TABLE 3:** Statistical analysis of differences between groups.

Assessment scale	T	df	*P*	Average difference	Standard error
FM_after	2.31	59.00	0.02	1.85	0.80
FM_before	–0.91	59.00	0.37	–0.56	0.62
FM_D	3.51	59.00	0.00	2.41	0.69
wolf_after	4.02	59.00	0.00	2.21	0.55
wolf_before	0.11	59.00	0.91	0.03	0.29
wolf_D	3.98	51.00	0.00	2.18	0.55
ARAT_after	1.27	59.00	0.21	1.60	1.26
ARAT_before	0.34	59.00	0.74	0.39	1.15
ARAT_D	3.01	59.00	0.00	1.21	0.40

*Fugl-Meyer test before and after (FM_before, FM_after), Wolf test before and after (lM_before, lM_after), ARAT test before and after (ARAT_before, ARAT_after). FM_D represents the difference between the scores of FM scale after intervention and before intervention. Wolf_D and ARAT_D scales are expressed in the same way.*

**TABLE 4:** Statistical analysis of differences before and after.

Group	classification	Average	*T*	df	*P*
A	FM_before and FM_after	−9.63	−18.15	29	0.00
	wolf_before and wolf_after	−5.47	−12.04	29	0.00
	ARAT_before and ARAT_after	−5.47	−16.50	29	0.00
B	FM_before and FM_after	−7.22	−16.45	30	0.00
	wolf_before and wolf_after	−3.29	−10.79	30	0.00
	ARAT_before and ARAT_after	−4.25	−18.38	30	0.00

*A stands for experimental group A, FM_ before and FM_ Afte indicates the result of comparing the difference before andafter using FM scale score. Other evaluation criteria are expressed in the same way.*

It can be seen from the table that there are significant differences between group A with “peripheral central peripheral” loop intervention and group B with manual energy robot assisted treatment, indicating that the two experimental paradigms have certain rehabilitation effects after intervention. The comparison results between groups can show that there is no difference between group A and group B before the intervention, and the prognosis and rehabilitation effect of group A is significantly better than that of group B.

**FIGURE 7:**
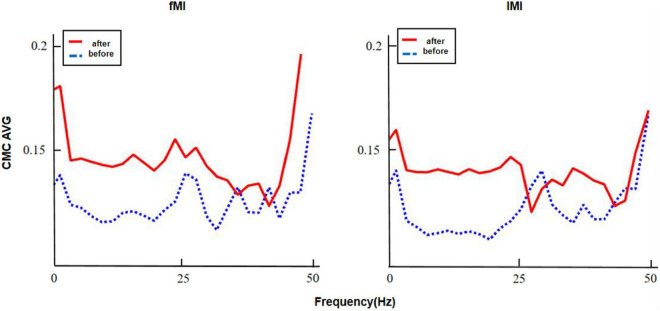
Mean value of cortical muscle coupling of 5 subjects before and after intervention.

We superimposed and averaged the CMC values on the frontoparietal cognitive area (fMl) and contralateral motor cortex area (lMl) of five subjects during the stable maintenance period. The results are shown in the figure. It can be seen from Figure 7 and Table 5 that the CMC coupling strength of patients is still at a low level (CMC < 0.2). Comparing the fMl region and lMl region before and after the intervention, it can be found that the CMC coupling of patients after the intervention is significantly improved in the beta band, but not in the gamma band.

**TABLE 5:** Statistical analysis of cmc value in frequency division.

Area	Band	Mean differences	*T*	df	*P*
fMl	Beta	–0.024	–4.23	4	0.013
	Gamma	0.087	0.84	4	0.449
lMl	Beta	–0.127	–29.793	4	0.00
	Gamma	–0.003	–1.58	4	0.19

*fMl and lMl represent frontal parietal lobe cognitive region and contralateral motor cortex region, respectively.*

Then we statistically analyzed the frequency bands of CMC values in the frontal parietal cognitive region and contralateral motor cortex. The statistical results are shown in the Table 4. In fMl area, the brain muscle coupling of beta band was significantly improved after patient intervention, and in lMl area, the brain muscle coupling of beta band was significantly improved after patient intervention. There was no significant difference before and after gamma band intervention.

## Discussion

By comparing the distribution characteristics of EEG power spectrum during MI and MI-SSVEP motor imagination tasks, we found that the right-hand motor imagination task induced by visual SSVEP stimulation can cause greater power response in the left motor cortex (lM) and occipital visual cortex (oM). The phenomenon of occipital lobe (oM) region is consistent with the experimental expectation and the previous research results on motor imagination task. At the same time, the power intensity of left and right motor cortex, occipital visual cortex and prefrontal cognitive cortex of MI and MI-SSVEP were compared. It was found that the activation intensity of occipital visual cortex and prefrontal cognitive cortex stimulated by MI-SSVEP was significantly higher than that of traditional motor imagination task. Mcgeady et al. (2019) studied a hybrid brain computer interface system. They tried to infer the effect of measuring multiple brain signals in motor imagination (MI) tasks. In addition to sensory motor rhythms (SMRs), they also introduced steady-state visual evoked potentials (SSVEP) to obtain additional information related to user intention. Their research results show that the hybrid brain computer interface with SSVEP has significantly improved the classification accuracy, but similar to the research of Jochunsen et al. they cannot obtain the internal neural mechanism of BCI induced neural plasticity from statistical experiments (Jochumsen et al., 2015). According to our existing research results, we infer that SSVEP visual stimulation under MI-SSVEP stimulation can enhance the activation of occipital cortex and motor imagination tasks. At the same time, in our experiment, we also found that the prefrontal cognitive cortex was always activated at a high level. Therefore, we speculate that visual stroboscopic can enhance the activation of frontal parietal lobe in motor imagination task by affecting the connection between occipital lobe and cognitive cortex.

Most neurological diseases have adverse effects on cognitive and motor functions. These injuries will not only change the measured values of EEG power spectrum and event-related potential, but also affect the consistency of EEG amplitude and phase (ITLC / ITPC) (Rosenblum et al., 2000). Therefore, observing ITLC / ITPC can reflect the ability of neural response and timely synchronization of related events (Makeig et al., 2004), so as to optimize information processing (Palva, 2005). In this study, we found the phase / linear consistency of motor imagination evoked potentials in different brain regions and different frequency bands under visual guidance. At the same time, there is a certain regularity in the distribution of phase / linear consistency between motor imagination tasks stimulated by MI-SSVEP and those without stroboscopic visual MI stimulation. The results show that: (1) the consistency change caused by motor imagination has an obvious gradual process in time distribution, which shows that the “coordination” between brain neurons can be improved after the start of motor imagination task, and this “coordination” represents the improvement of brain efficiency in information processing. From the experimental results, it can be concluded that the improvement of efficiency is a gradual process. The brain needs a “pre-treatment” process to adapt to the activation effect of motor imagination task; (2) When subjects performed motor imagination tasks under MI-SSVEP stimulation, there was a high consistency distribution in the beta band of frontal parietal lobe within 3–6 s, but this feature did not appear in the MI stimulation group. At the same time, there was a significant high consistency distribution in the alpha band of MI-SSVEP stimulation within 4–6 s in the occipital region. This shows that MI-SSVEP stimulation can more effectively cause the information processing efficiency of frontoparietal cortex, and the power of this region is also significantly improved. The consistency difference of frontal parietal lobe region shows that the motor imagination task guided by stroboscopic stimulation is a complex feedback process, which may be related to the activation of occipital lobe; (3) At the same time, in the LM region, both MI-SSVEP stimulation and Mi stimulation have a high consistency distribution in the beta frequency band within 3–4 s, and then the MI-SSVEP consistency distribution shifts to the low gamma frequency band. Papenberg et al. (2016) studied the characteristics of linear consistency distribution in different frequency bands. They believe that the linear consistency of low gamma frequency band is related to the improvement of motor function, The improvement of consistency shows the change of response time variability of motor cortex to motor task in motor task. The faster the response time, the higher the consistency. That is, stroboscopic stimulation enhances motor imagination response (Papenberg et al., 2013).

Convergent cross mapping (Ye et al., 2015) based on EEG data is a new computational method to study the connectivity of human brain network (Clark et al., 2015). In our study, the visual evoked motor imagination task under stroboscopic stimulation was introduced. We found that the unidirectional causal relationship between motor sensory cortex and EMG channels increased significantly during MI-SSVEP stimulation compared with visual evoked motor imagination alone. We believe that in the current study, the causal difference between occipital cortex and parietal cognitive cortex reflects the interactive intervention mechanism between occipital cortex and parietal cognitive cortex in MI-SSVEP induced motor imagination tasks.

Combined with previous studies and the findings on the linear consistency between groups and CCM causality in this chapter’s experiments, we speculate that the principle of this intervention may come from two aspects: (1) stroboscopic motion picture stimulation improves the subjects’ attention concentration in the process of motion observation and motion imagination; (2) Similar to the process of human brain processing film, human photosensitive system can distinguish up to 48 flashes per second. If the frequency continues to increase, the conversion between light and dark will not be detected (Watson and Ahumada, 1985), For motion pictures, humans will keep the images they see for 100–400 ms. Therefore, when people see motion pictures with a frame rate of more than 15, they will think that they are continuous motion movements (Crick et al., 1981). In our experiment, the frame rate of MI-SSVEP is 10, so it will lead to an “involuntary” motion imagination process in the cerebral cognitive cortex to supplement the missing pictures. This “involuntary” motor imagination process may lead to the cognitive cortex group not only in the process of motor imagination, but also constantly “waiting” or “grasping” the “new” picture information coming from the occipital visual cortex. This process of “waiting” or “grasping” directly leads to the enhancement of causality between the two cortexes.

In the clinical study, we used two different intervention methods to treat 84 stroke patients. From the clinical experiment, we found that: (1) after two weeks of intervention, the traditional robot assisted rehabilitation therapy and “MI-SSVEP” intervention can effectively improve the scores of multiple scales; (2) Comparing the therapeutic effects of the two intervention paradigms, “MI-SSVEP” intervention can more effectively improve the rehabilitation effect of patient intervention. (3) “MI-SSVEP” intervention can effectively improve the brain muscle coupling effect of beta band in patients, indicating that it does have a neural mechanism to improve the effect of rehabilitation treatment. The significant effect of robot assisted therapy on clinical intervention is completely within the expected range. A large number of previous studies on robot assisted therapy have proved the effectiveness of this therapy (Rodgers et al., 2017). We analyzed deeply the scores of patients with “peripheral central peripheral” motor loop nerve intervention treatment scheme integrated with peripheral feedback in Fugl-Meyer, Wolf and ARAT evaluation scales. It was found that compared with traditional robot assisted treatment, patients with new treatment scheme had relatively high scores in fine motor and muscle tension. Combined with the follow-up visit to patients in clinical experiments, we believe that this improvement is mainly due to the following reasons: (1) in the process of patient intervention, compared with the traditional robot assisted passive treatment, MI-SSVEP intervention paradigm can mobilize the attention concentration of patients’ rehabilitation and make patients more focused during training time. (2) The real-time effect of visual stimulus input can change the CMC distribution of primary motor somatosensory cortex and contralateral motor cortex. This effect not only has real-time effect, but also has prognostic effect. (3) MI-SSVEP guided motor imagination task can more effectively improve the intervention effect of motor imagination in the training process, and can stimulate motor imagination activities more directly.

## Data Availability Statement

The original contributions presented in the study are included in the article/supplementary material, further inquiries can be directed to the corresponding author/s.

## Ethics Statement

The studies involving human participants were reviewed and approved by the Ethics Committee of Xi’an Jiaotong University. The patients/participants provided their written informed consent to participate in this study. Written informed consent was obtained from the individual(s) for the publication of any potentially identifiable images or data included in this article.

## Author Contributions

LL participated to study design, data collection and analysis, and manuscript writing. YZ participated to study design, data collection and analysis, and manuscript definition. LH participated to data analysis. JZ participated in the data collection. JW and TL participated to study design. All authors read and approved the final manuscript.

## Conflict of Interest

The authors declare that the research was conducted in the absence of any commercial or financial relationships that could be construed as a potential conflict of interest.

## Publisher’s Note

All claims expressed in this article are solely those of the authors and do not necessarily represent those of their affiliated organizations, or those of the publisher, the editors and the reviewers. Any product that may be evaluated in this article, or claim that may be made by its manufacturer, is not guaranteed or endorsed by the publisher.
